# Supplementary material for: MaxQuant Software for Ion Mobility Enhanced Shotgun Proteomics
Source: Mol Cell Proteomics. 2020 Mar 10;19(6):1058–69. doi: 10.1074/mcp.TIR119.001720 (PMC7261821; doi:10.1074/mcp.TIR119.001720)
Supplement: Supplementary Figures [file 155063_1_supp_468101_q4z5cq.pdf]

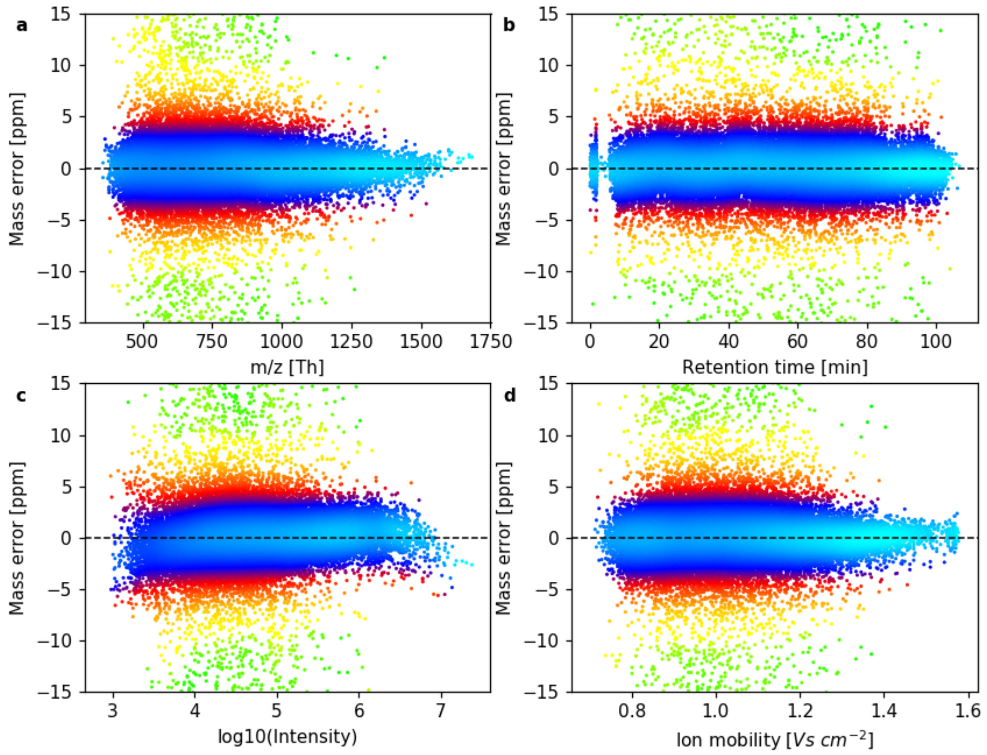

Supplementary Figure 1. Mass error after recalibration. **a.-d.** Residual mass errors after complete recalibration, showing the dependence of the residual mass error on *m*/*z* (**a.**), retention time (**b.**), logarithm of the peak intensity (**c.**) and ion mobility (**d.**). Colors reflect the density of data points.
