# Supplementary figures and images for: MaxQuant Software for Ion Mobility Enhanced Shotgun Proteomics
Source: Mol Cell Proteomics. 2020 Mar 10;19(6):1058–69. doi: 10.1074/mcp.TIR119.001720 (PMC7261821; doi:10.1074/mcp.TIR119.001720)

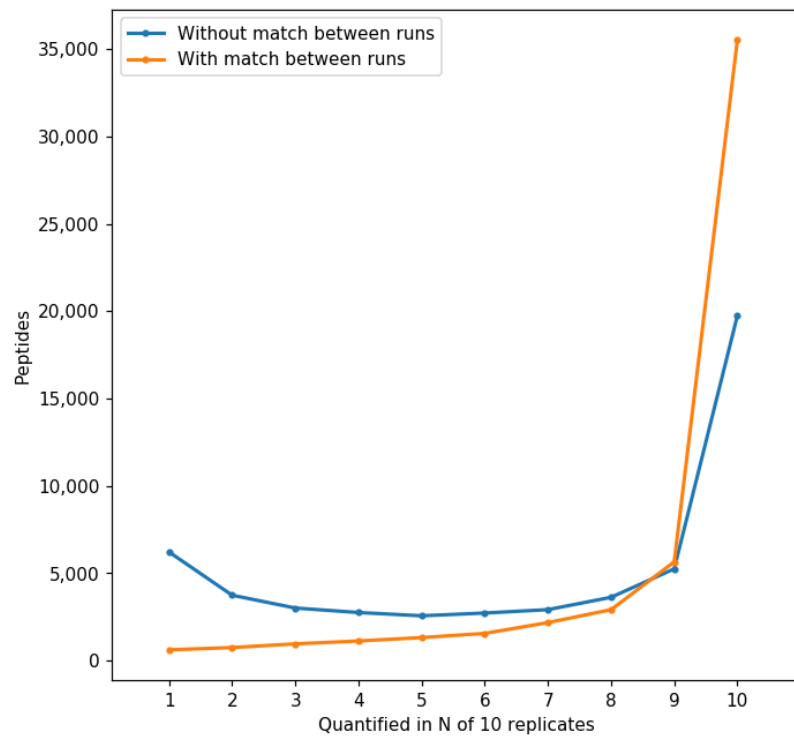

Supplement: Supplementary Fig. 2 [file 155063_1_supp_468175_q4z9zx.pdf]
